# Supplementary material for: Targeting the BRD4/FOXO3a/CDK6 axis sensitizes AKT inhibition in luminal breast cancer
Source: Nat Commun. 2018 Dec 5;9:5200. doi: 10.1038/s41467-018-07258-y (PMC6281582; doi:10.1038/s41467-018-07258-y)
Supplement: Supplementary file 1 — Supplementary Information [file 41467_2018_7258_MOESM1_ESM.pdf]

Targeting the BRD4/FOXO3a/CDK6 axis Sensitizes AKT Inhibition in Luminal Breast Cancer

Jingyi Liu[1,2,3], Zhibing Duan[1,2], Weijie Guo[1,2], Lei Zeng[4,5], Yadi Wu[2,6], Yule Chen[1,2], Fang Tai[7], Yifan Wang[1,2], Yiwei Lin[1,2], Qiang Zhang[4,5], Yanling He[7], Jiong Deng[8], Rachel L. Stewart[2], Chi Wang[2], Pengnian Charles Lin[3], Saghi Ghaffari[9], B. Mark Evers[2,10], Suling Liu[11*], Ming-Ming Zhou[4*], Binhua P. Zhou[1,2*], Jian Shi[1,2,7*]

Supplementary Information:

Supplementary Figure 1
Supplementary Figure 2
Supplementary Figure 3
Supplementary Figure 4
Supplementary Figure 5
Supplementary Figure 6
Supplementary Table 1
Supplementary Table 2

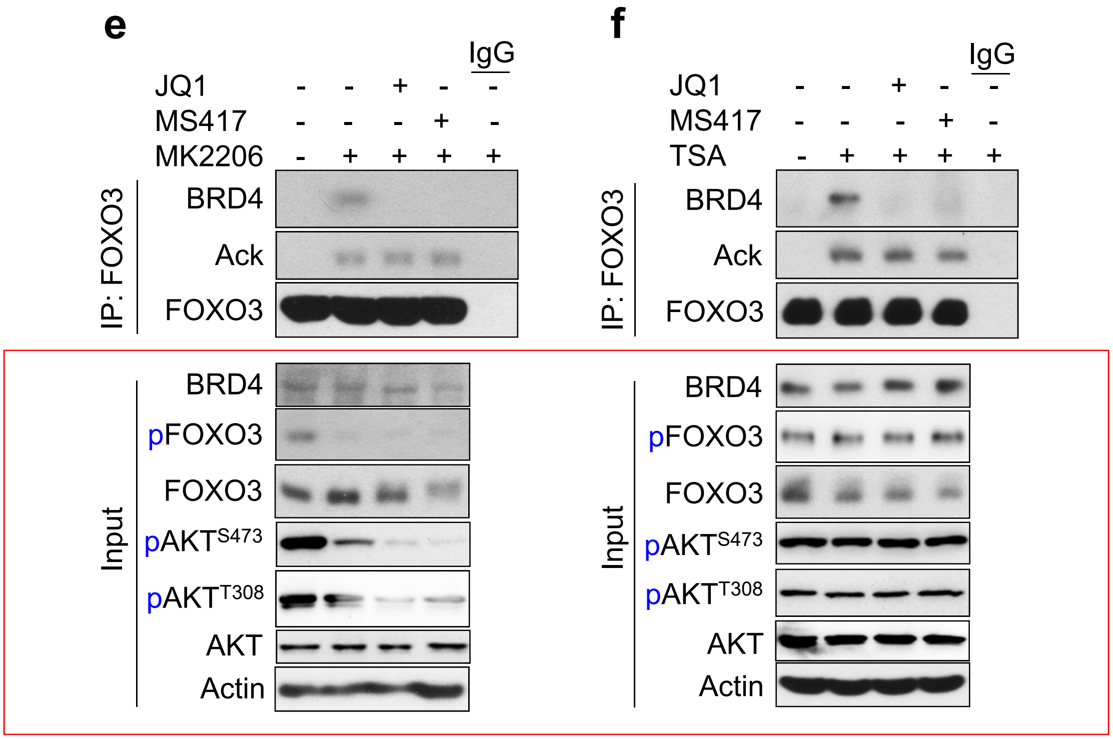

Supplementary Figure 1

Western blots showing expression of various molecules in input lysates of Figs 2e and 2f.

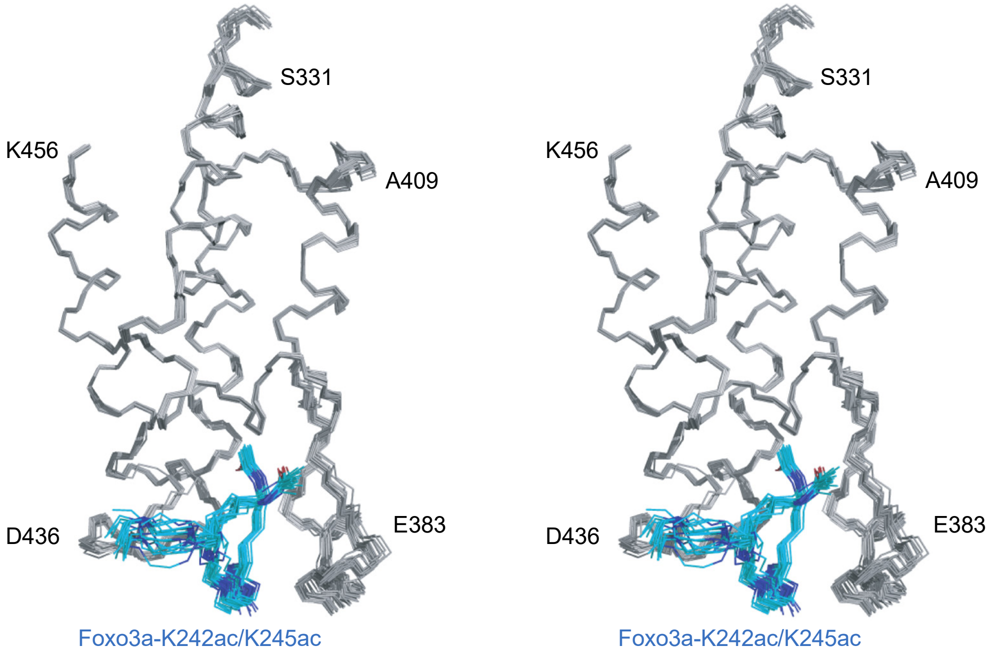

Supplementary Figure 2

The 3D structure of the BRD4 BD2 in complex with a Foxo3a-K242ac/K245ac peptide (residues 352-455) is shown as stereo-view of the backbone atoms (N, Cα and C') of 20 superimposed NMR structures of the complexes.

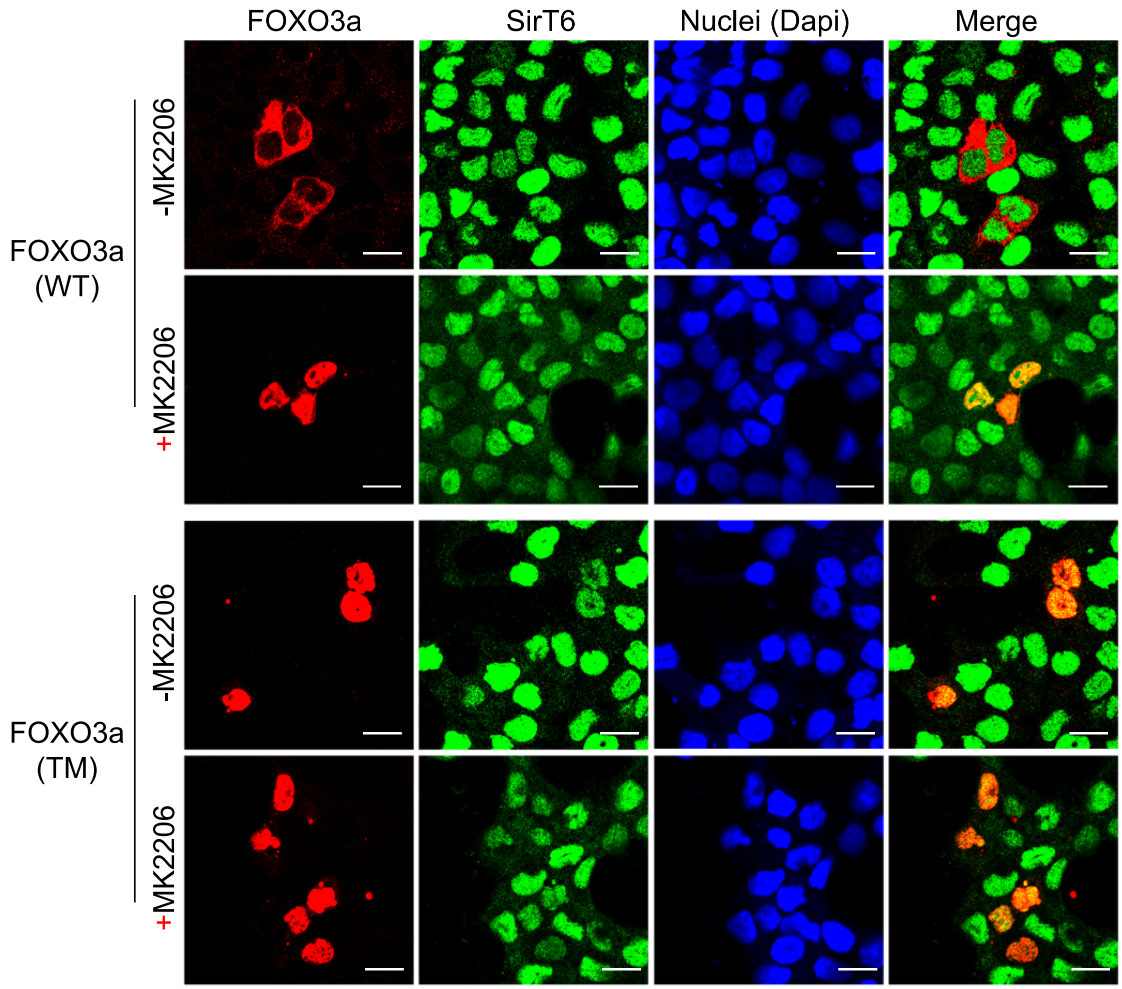

Supplementary Figure 3
HA-tagged WT- or TM-FOXO3a was expressed in T47D cells treated with or without 1 μM MK2206 for 6 h. The cellular localization of FOXO3a (red) and endogenous SirT6 (green) were analyzed by immunofluorescent staining. Nuclei stained with DAPI (blue). Scale bar, 20 μM.

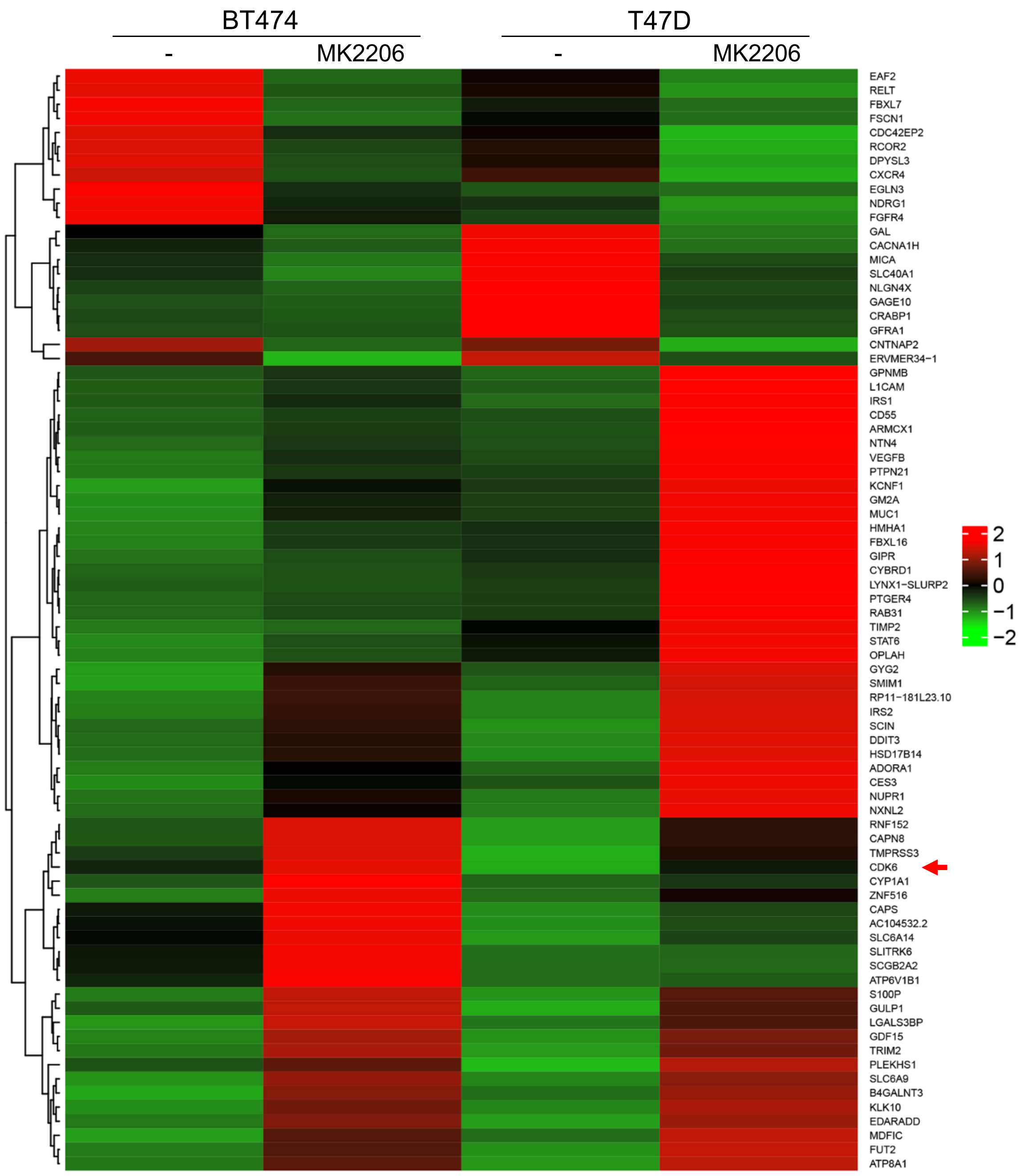

Supplementary Figure 4
Large heat-map showing the differentially expressed genes from Fig 5b.

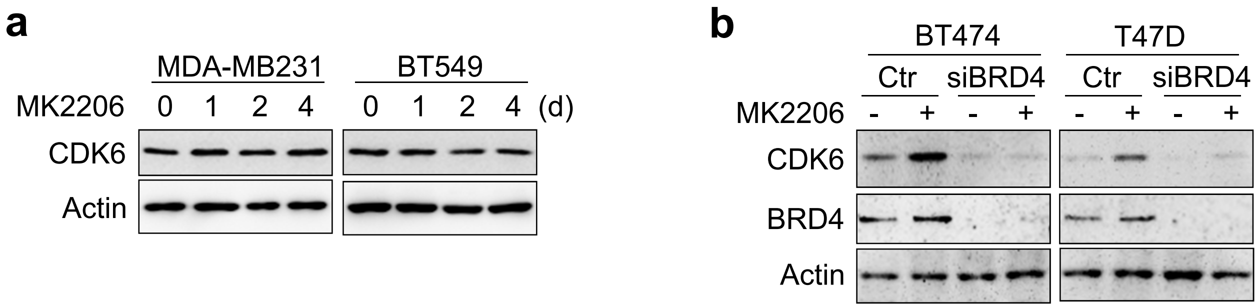

Supplementary Figure 5

(a) MDA-MB231 and BT549 cells were treated with 1 μM MK2206 for different time intervals, expression of CDK6 was analyzed by western blot.

(b) Induction of CDK6 by MK2206 (1 μM) was analyzed by western blot in BT474 and T47D cells with or without BRD4-knockdown.

Supplementary Figure 6. Full scans of western blots images.

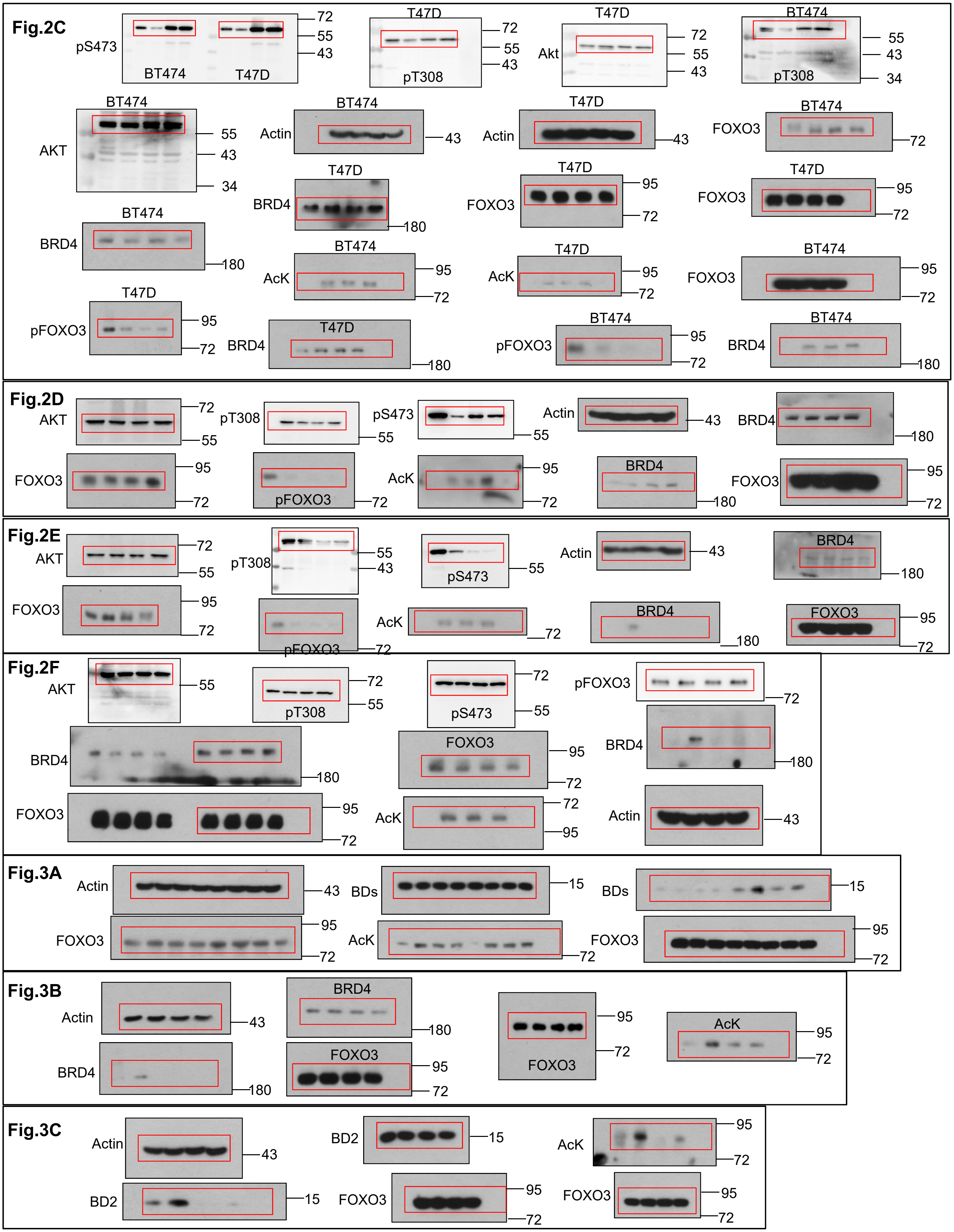

Fig.3F

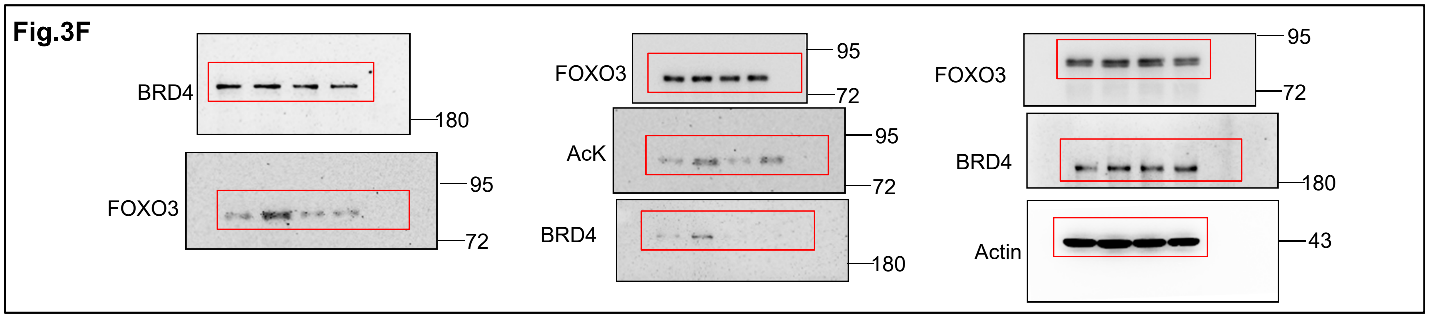

Fig.4A

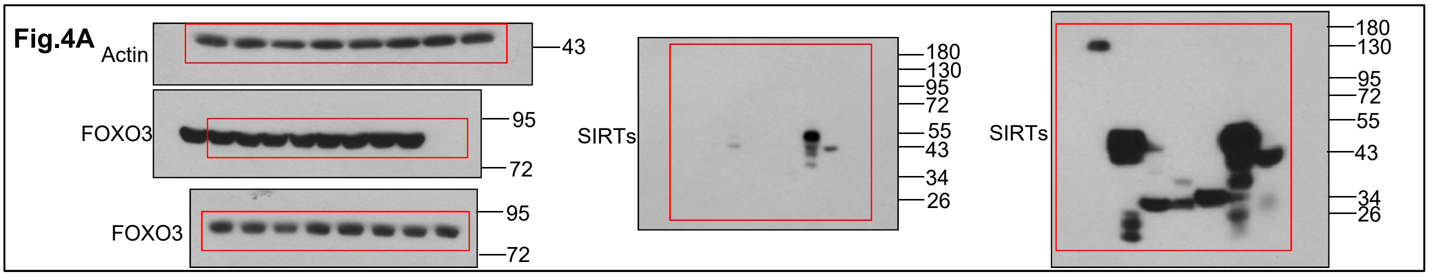

Fig.4B

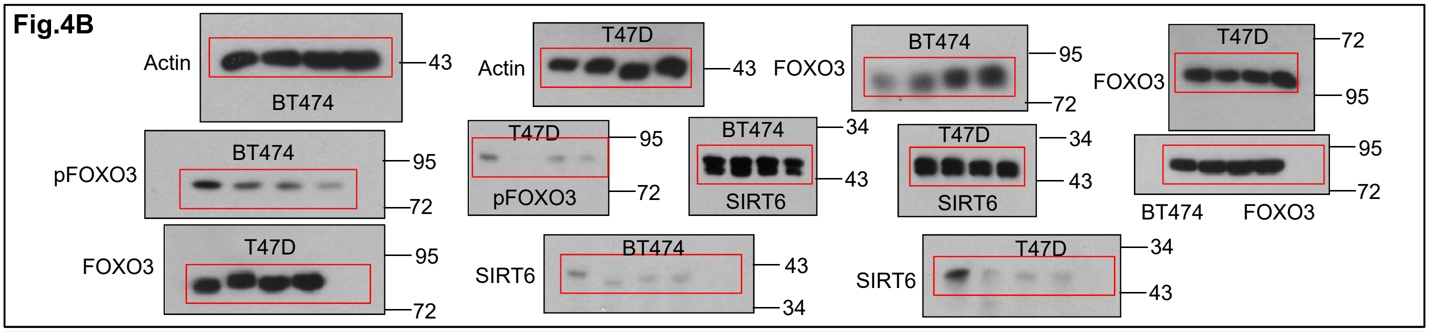

Fig.4C

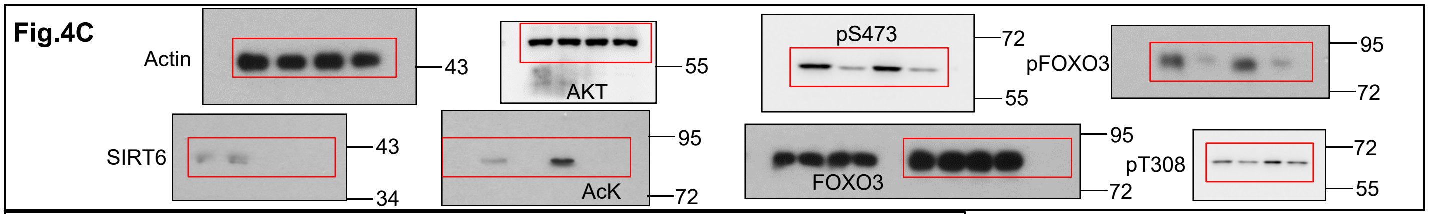

Fig.4D

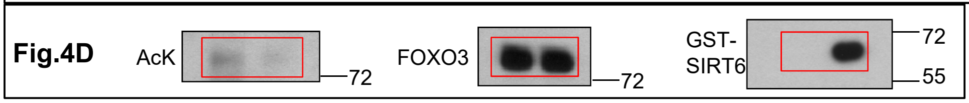

Fig.4E

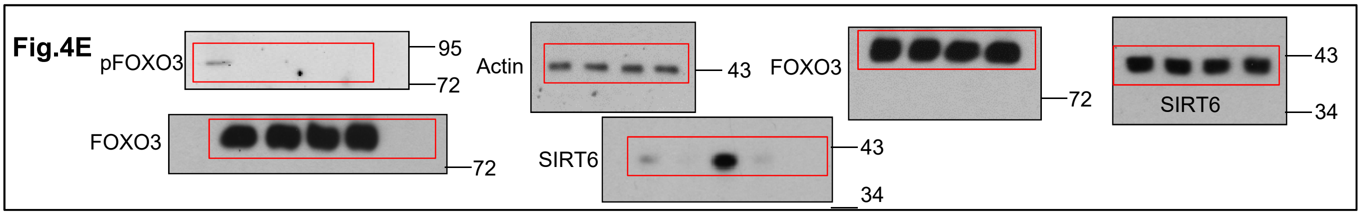

Fig.4F

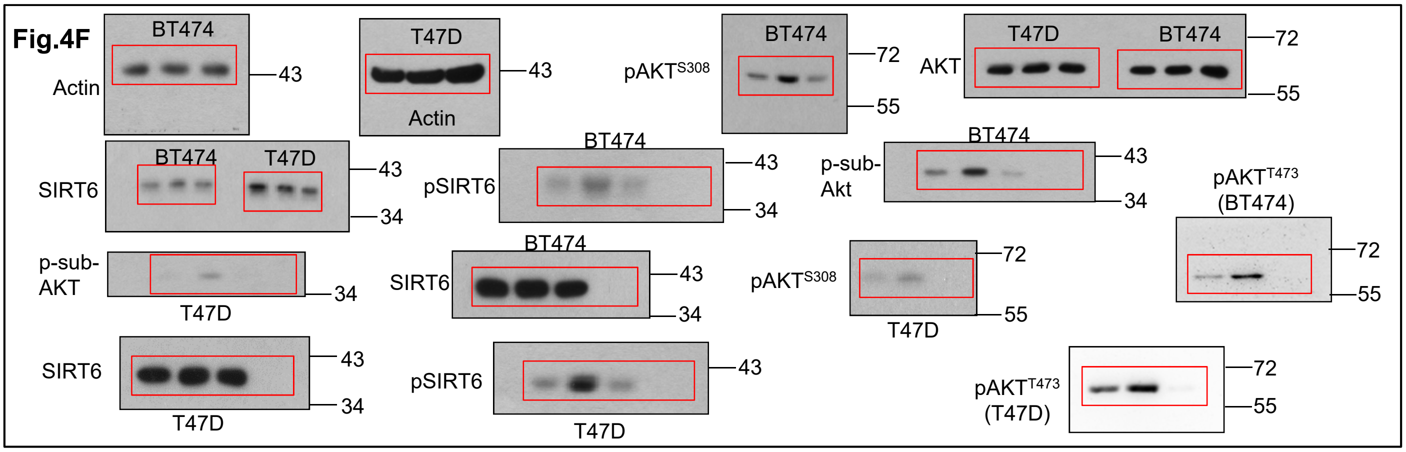

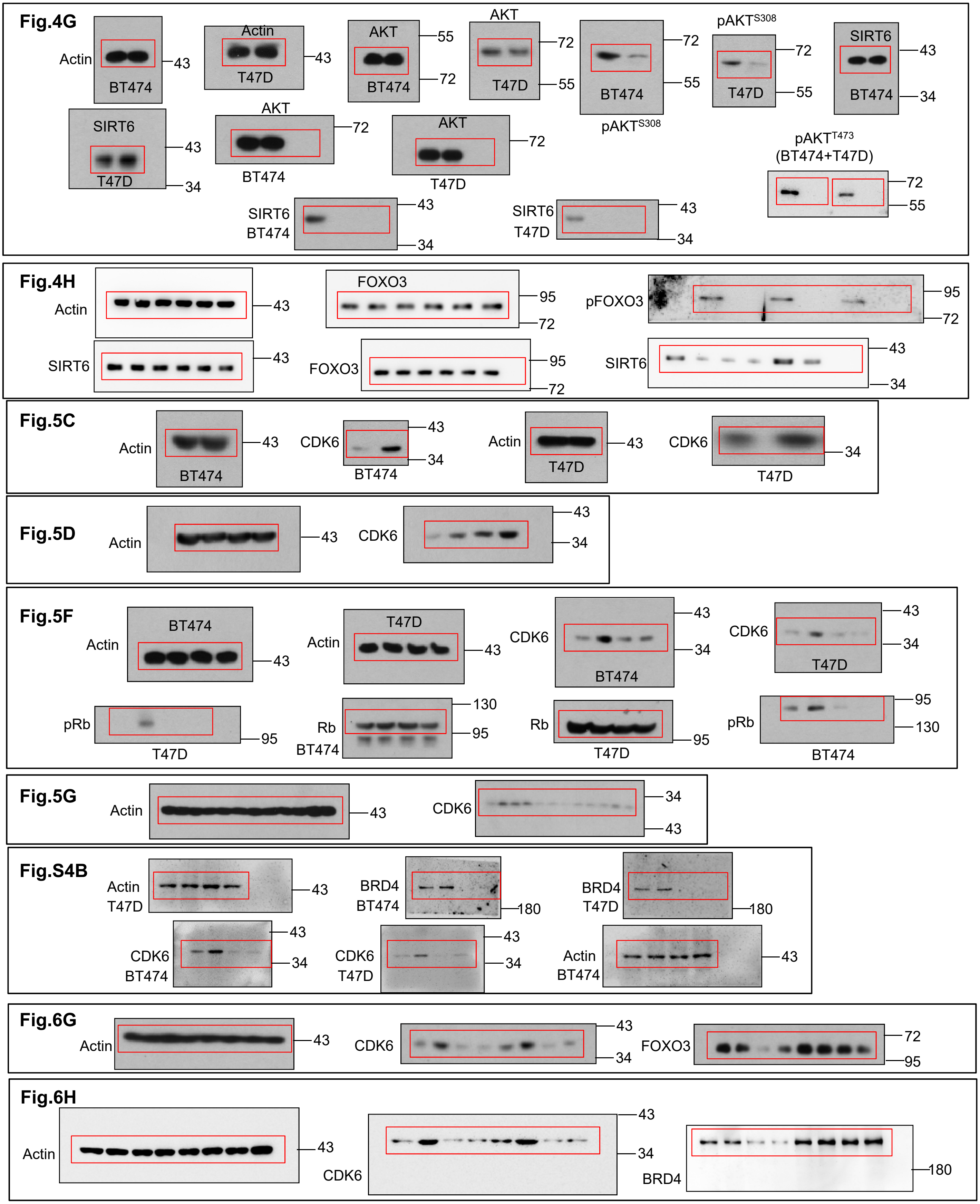

Fig.4G
Actin
BT474
43
Actin
T47D
43
AKT
BT474
55
72
AKT
T47D
72
55
BT474
72
55
pAKTS308
pAKTS308
T47D
72
55
SIRT6
BT474
43
34
SIRT6
T47D
43
34
AKT
BT474
72
AKT
T47D
72
pAKTT473
(BT474+T47D)
72
55
SIRT6
BT474
43
34
SIRT6
T47D
43
34
Fig.4H
Actin
43
FOXO3
95
72
pFOXO3
95
72
SIRT6
43
FOXO3
95
72
SIRT6
43
34
Fig.5C
Actin
BT474
43
CDK6
BT474
43
34
Actin
T47D
43
CDK6
T47D
34
Fig.5D
Actin
43
CDK6
43
34
Fig.5F
Actin
BT474
43
Actin
T47D
43
CDK6
BT474
43
34
CDK6
T47D
43
34
pRb
T47D
95
Rb
BT474
130
95
Rb
T47D
95
pRb
BT474
95
130
Fig.5G
Actin
43
CDK6
34
43
Fig.S4B
Actin
T47D
43
BRD4
BT474
180
BRD4
T47D
180
CDK6
BT474
43
34
CDK6
T47D
43
34
Actin
BT474
43
Fig.6G
Actin
43
CDK6
43
34
FOXO3
72
95
Fig.6H
Actin
43
CDK6
43
34
BRD4
180

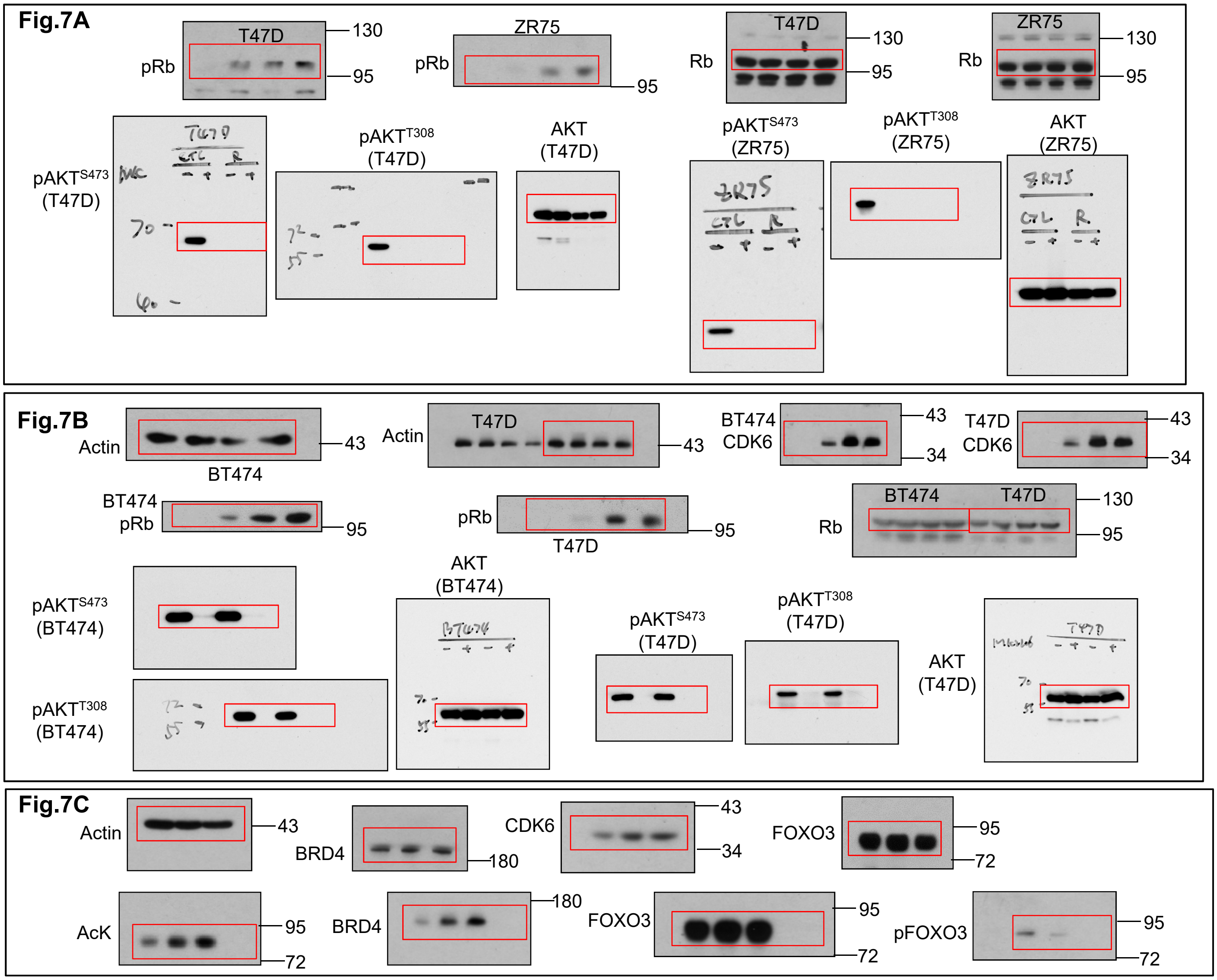
Fig.7A
T47D
pRb
130
95
ZR75
pRb
95
T47D
Rb
130
95
ZR75
Rb
130
95
pAKT^S473 (T47D)
pAKT^T308 (T47D)
AKT (T47D)
pAKT^S473 (ZR75)
pAKT^T308 (ZR75)
AKT (ZR75)
Fig.7B
Actin
43
BT474
Actin
T47D
43
BT474 CDK6
43
34
T47D CDK6
43
34
BT474 pRb
95
pRb
95
T47D
Rb
BT474
T47D
130
95
pAKT^S473 (BT474)
AKT (BT474)
pAKT^S473 (T47D)
pAKT^T308 (T47D)
AKT (T47D)
pAKT^T308 (BT474)
Fig.7C
Actin
43
BRD4
180
CDK6
43
34
FOXO3
95
72
AcK
95
72
BRD4
180
FOXO3
95
72
pFOXO3
95
72

Supplementary Table 1: Antibodies used in the study

Antibody	Vendors	Catalogue No	Applications	Dilution
Akt	CST	2938	WB	1:1000
pAkt (T308)	CST	2965	WB	1:1000
pAkt (S473)	CST	4060	WB	1:1000
CDK6	CST	13331	WB	1:1000
FOXO3	CST	12829	WB	1:1000
FOXO3	CST	12829	IP	1:250
FOXO3	CST	12829	IF	1:100
BRD4	CST	13440	WB	1:1000
BRD4	CST	13440	IP	1:250
SIRT6	CST	12486	WB	1:1000
Phospho-Akt sub	CST	9614	WB	1:1000
Acetyl-lysine	CST	9441	WB	1:1000
Rb	CST	9313	WB	1:1000
pRb	CST	8180	WB	1:1000
HA	Roche	11867423001	WB	1:5000
HA	Roche	11867423001	IP	1:500
Flag	Sigma	F3165	WB	1:5000
Flag	Sigma	F3165	IP	1:500
Actin	Sigma	A5441	WB	1:10,000

Supplementary Table 1. Summary of Statistics of NMR Structures of the BRD4-BD2/FOXO3a Peptide Complex

	BRD4-BD2/FOXO3a
NMR distance and dihedral constraints	
Distance constraints	
Total NOE	2157
Intra-residue	623
Inter-residue	1534
Sequential ($\|i - j\| = 1$)	453
Medium-range ($\|i - j\| < 4$)	504
Long-range ($\|i – j\| > 5$)	577
Inter-molecular	137
Hydrogen bonds	56
Total dihedral angle restraints	
Φ angle	97
Ψ angle	97
Ramachandran Map Analysis (%)	
Most favored regions	98.8
Additional allowed regions	1.2
Generously allowed regions	0.0
Disallowed regions	0.0
Structure statistics	
Violations (mean ± S.D.)	
Distance constraints (Å)	0.042 ± 0.0053
Dihedral angle constraints (º)	0.18 ± 0.11
Max. dihedral angle violation (º)	0.46
Max. distance constraint violation (Å)	0.054
Deviations from idealized geometry	
Bond lengths (Å)	0.0040 ± 0.00014
Bond angles (º)	0.58 ± 0.017
Impropers (º)	1.3 ± 0.043
Average pairwise r.m.s. deviation (Å)	
Heavy	0.62 ± 0.061
Backbone	0.27 ± 0.045

a. Procheck residue numbers are 348-380, 397-409, 411-436, and 437-456.
b. The residue number range used in full molecule root-mean-square (r.m.s.) deviation calculations consists of 352-455.
c. Pairwise r.m.s. deviation was calculated among top 20/200 lowest energy structures.
